# A novel nomogram based on body composition and nutritional indicators to predict the prognosis of patients with muscle‐invasive bladder cancer undergoing radical cystectomy

**DOI:** 10.1002/cam4.6712

**Published:** 2023-11-16

**Authors:** Xin Zhong, Yunzhe Pan, Kang Wu, Langkun Wang, Peng Dou, Ping Tan, Peng Zhang, Xiang Li

**Affiliations:** ^1^ Department of Urology Institute of Urology, West China Hospital of Sichuan University Chengdu Sichuan China; ^2^ Department of Urology Chengdu Second People's Hospital Chengdu Sichuan China

**Keywords:** body composition, MIBC, nomogram, nutritional indicators, prognosis

## Abstract

**Objective:**

To investigate the prognostic significance of body composition and nutritional indicators in patients undergoing radical cystectomy with muscle‐invasive bladder cancer (MIBC) and to develop a novel nomogram that accurately predicts overall survival (OS).

**Methods:**

From December 2010 to December 2020, we retrospectively collected clinical and pathological data from 373 MIBC patients who underwent radical cystectomy at our hospital. Preoperative computed tomography (CT) images were used to measure the skeletal muscle index (SMI), subcutaneous adipose index (SAI), visceral adipose index (VAI), skeletal muscle density (SMD), subcutaneous adipose density (SAD), visceral adipose density (VAD), and visceral adipose to subcutaneous adipose area ratio (VSR). The clinicopathological characteristics were evaluated using LASSO regression and multivariate Cox regression, and a nomogram was constructed to predict 1‐, 3‐, and 5‐year overall survival. The concordance index (C‐index), time‐dependent receiver operating characteristic curves (t‐ROC), calibration curve, and decision curve analysis (DCA) were used to assess the discriminative ability, calibration, and clinical practicality of the nomogram.

**Results:**

Multivariate analyses demonstrated that pT stage, lymph node status, LVI, SMD, and prognostic nutritional index (PNI) are independent prognostic factors for OS. Additionally, a nomogram was created. The nomogram's C‐index was 0.714 (95% CI: 0.695–0.733). The area under the t‐ROC curve of 1‐, 3‐, and 5‐year survival corresponding to the model was 0.726, 0.788, and 0.785, respectively. The calibration curve demonstrated excellent agreement between the predicted and observed outcomes. The DCA revealed that patients with MIBC could benefit from the nomogram.

**Conclusion:**

Based on body composition and nutritional indicators, we developed a novel nomogram with excellent predictive accuracy and reliability for predicting the prognosis of MIBC patients undergoing RC.

## 1 INTRODUCTION

Bladder cancer is common and fatal on a global scale. According to a recent report by Sung H. et al., the incidence of bladder cancer is tenth worldwide, with an estimated 573,000 new cases and 213,000 fatalities annually.^1^ On the basis of the extent of tumor invasion, bladder cancer can be divided into non‐muscle‐invasive bladder cancer (NMIBC) and muscle‐invasive bladder cancer (MIBC). Approximately 25% of patients were initially diagnosed with MIBC, and 20% of NMIBC patients progressed to MIBC within 5 years.^2^, ^3^ The recommended treatment of choice for MIBC is radical cystectomy (RC) in conjunction with pelvic lymph node dissection.^3^ Nevertheless, 50% of MIBC patients will experience distant metastasis after RC, and the five‐year overall survival (OS) for MIBC ranges from 46% to 63%.^4^, ^5^

The TNM staging system of the American Joint Committee on Cancer (AJCC) is extensively utilized to predict the prognosis of MIBC patients.^6^ Predicting postoperative OS, however, still has limitations. Even among those in the same TNM stage, the prognosis for patients with MIBC can vary substantially. Nomograms are regarded as an accurate method for predicting individual MIBC survival following RC. Moreover, the nomogram is more predictive than the AJCC stage classification alone.

Body composition analysis focuses primarily on adipose and skeletal muscle tissue. Studies have shown that the content and proportion of visceral fat, subcutaneous fat, and skeletal muscle are associated with the clinical outcome of patients with malignant tumors, and they have a substantial impact on the occurrence, progression, and prognosis of the disease.^7^ Multiple CT‐based body composition parameters have been utilized to predict the prognosis of patients with colorectal and gastric cancer, but comparable MIBC studies are lacking.^7^, ^8^, ^9^ The prognostic nutritional index (PNI) is computed by merging the levels of serum albumin and lymphocyte count, serving as an assessment tool for the nutritional and immune conditions of individuals with tumors.^10^ PNI has been proven to be linked to the prognosis of multiple malignancies, including esophageal cancer, gastric cancer, and liver cancer.^11^, ^12^, ^13^ The controlling nutritional status (CONUT) score is a scoring system for evaluating patients' nutritional status based on serum albumin, lymphocyte count, and total cholesterol.^14^ Compared with PNI, the CONUT score incorporates total cholesterol, an essential component of the cell membrane, and its serum concentration level is associated with the development of tumors. Multiple research studies have shown that the CONUT score is a dependable marker for the postoperative survival of individuals diagnosed with gastrointestinal tumors.^14^, ^15^ Nonetheless, the correlation between PNI, COUNT score, and the prognosis of MIBC patients undergoing RC is still not well understood.

The objective of this study was to create a prognostic model for the overall survival of MIBC patients who underwent RC by integrating preoperative nutritional and body composition indicators. By utilizing this nomogram, clinicians and patients can enhance their decision‐making regarding treatment and management by accessing more personalized prognoses.

## 2 MATERIALS AND METHODS

### 2.1 Participants in the study

We retrospectively obtained data from patients diagnosed with MIBC and treated with RC at West China Hospital from December 2010 to December 2020. The following are the criteria for exclusion: (1) lack of preoperative CT images and blood examination data; (2) loss to follow‐up; (3) lack of TNM stage; (4) metastasis prior to surgery; (5) nonurothelial carcinoma; and (6) presence of other tumors. In the end, 337 patients with MIBC confirmed pathologically were included. All operations were carried out by experienced urological surgeons and in strict accordance with standard operating procedures.

The retrospective analysis followed the guidelines of the Declaration of Helsinki and received approval from the Ethics Committee of West China Hospital, Sichuan University.

### 2.2 Evaluation of body composition

**FIGURE 1:**
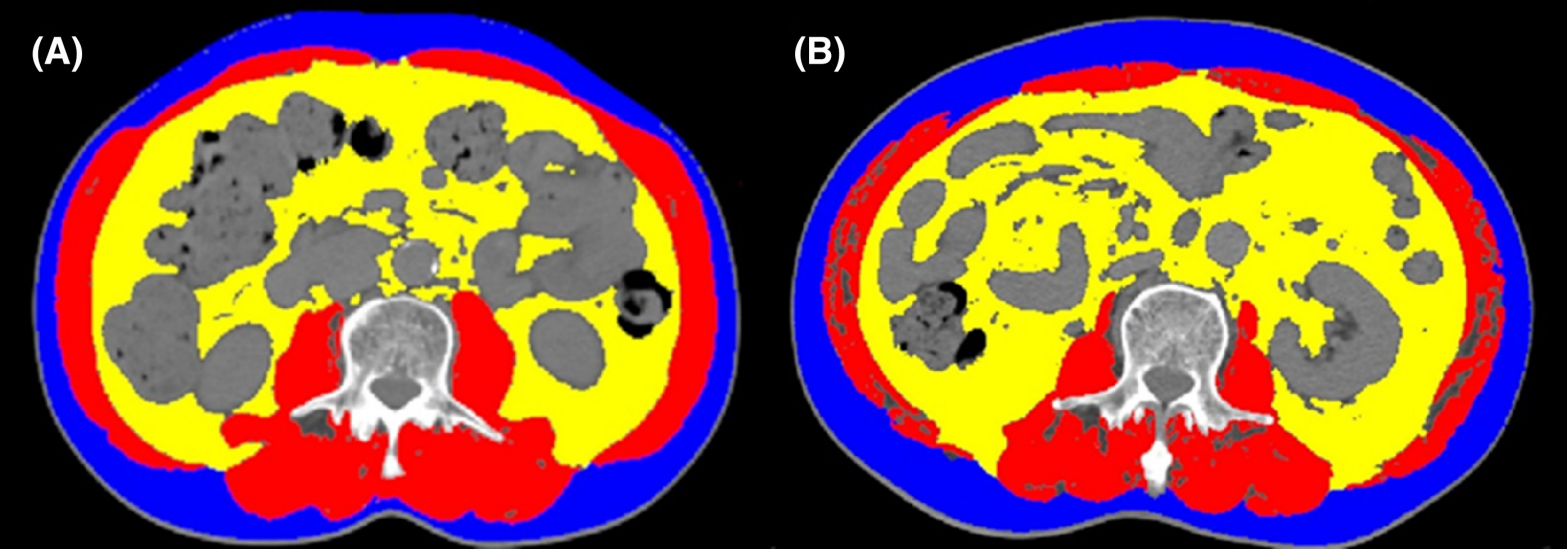
Examples of subcutaneous fat area (blue), visceral fat area (yellow), and skeletal muscle area (red) segmentations at the third lumbar vertebra from two different patients.

Every participant involved in the research underwent CT scans within a fortnight prior to the operation. For body composition analysis, a CT image of the third lumbar vertebra (L3) was chosen using Tomovision's SliceOmatic™ software (v5.0, Magog, Quebec, Canada). The following are the various tissue Hounsfield unit (HU) thresholds: (1) skeletal muscle ranges from −29 to +150 HU; (2) subcutaneous adipose tissue ranges from −190 to −30 HU; and (3) visceral adipose tissue ranges from −150 to −50 HU. Figure 1 illustrates the different body compositions. Two investigators were instructed to conduct the measurement independently to reduce bias.

The skeletal muscle index (SMI), subcutaneous adipose index (SAI), and visceral adipose index (VAI) were calculated using the normalized areas of skeletal muscle, subcutaneous adipose tissue, and visceral adipose tissue. The skeletal muscle density (SMD) is obtained by averaging the HU radiation density of the total cross‐sectional skeletal muscle based on preoperative CT. Similarly, subcutaneous adipose density (SAD) and visceral adipose density (VAD) were calculated based on preoperative CT. Visceral adiposity was measured using the ratio of visceral to subcutaneous adipose area (VSR).

### 2.3 PNI and CONUT score

Serum samples were collected and assayed within 1 week prior to operation. Using the following formula, the PNI was computed: serum albumin concentration (g/L) +5*lymphocyte count (/L). The CONUT score was derived from three common peripheral blood parameters (albumin, total lymphocyte count, and total cholesterol) (Table 1).

**TABLE 1:** The scoring system of CONUT score.

Parameter	None	Light	Moderate	Severe
Serum albumin (g/L)	≥35	30 ~ 34.9	25 ~ 29.9	<2.50
Score	0	2	4	6
Lymphocyte count (×10^9^/L)	≥1.60	1.20 ~ 1.59	0.80 ~ 1.19	<0.80
Score	0	1	2	3
Total cholesterol (mg/dL)	≥180	140 ~ 179	100 ~ 139	<100
Score	0	1	2	3

Abbreviation: COUNT, controlling nutritional status.

### 2.4 Covariates

The clinicopathological data collected in retrospect consisted of information on sex, age, body mass index (BMI), past occurrences of hypertension and diabetes, smoking habits, pT stage, size of the tumor, grade of the tumor, number of tumors, invasion of peripheral nerve, status of lymph nodes, presence of lymphovascular invasion (LVI), presence of carcinoma in situ (CIS), presence of variant histology, and existence of positive surgical margins (PSM). The TNM classification of the AJCC Staging Manual (8th edition) was utilized to stage all specimens, while the 1973 World Health Organization grading system was employed to grade them. Recorded information about the procedure included details on the surgical techniques used, the removal of pelvic lymph nodes, and the urinary diversion.

### 2.5 Construction of the nomogram

To screen out variables substantially associated with OS, univariate Cox regression analyses were performed initially. Variables with a *p* value <0.05 in the univariate analyses were chosen. The chosen variables were subsequently incorporated into the least absolute shrinkage and selection operator (LASSO) regression. The categorical variables were assigned dummy variables. To ensure the appropriateness of the tuning parameters (λ) for LASSO regression, cross‐validation was employed. LASSO regression was then used to identify the most significant variables. Finally, the above variables selected by LASSO regression were then subjected to multivariate Cox proportional hazards analyses; variables with *p* values <0.05 from multivariate analyses were incorporated into nomograms predicting the 1‐, 3‐, and 5‐year OS.

### 2.6 Statistical analysis

The statistical analyses were conducted with R (version 4.2.1). If the data were continuous and followed a normal distribution, the descriptive statistical analyses would show the mean ± standard deviation (SD); otherwise, the median (upper and lower quartiles) would be reported. For categorical data, the number (N) and percentage (%) were described. Depending on the characteristics of the data, differences between groups were evaluated using ANOVA, Pearson's χ^2^ test, Fisher's exact test, or the Kruskal–Wallis test. Using receiver operating characteristic (ROC) curves and the Youden Index, the optimal threshold value for the indicators of body composition was determined. The LASSO regression method was predominantly utilized to select potential predictive variables, resolve collinearity, and prevent overfitting. The Cox regression model was used to evaluate the correlation between risk factors and OS. Additionally, the prediction model was validated using the bootstrap resampling method. Kaplan–Meier analysis and the log‐rank test were used to evaluate the risk variables for OS, and the findings were expressed as hazard ratios (HRs) and associated 95% confidence intervals (CIs). The nomogram's calibration and discrimination were evaluated using the calibration curve and the area under the time‐dependent receiver operating characteristic curve (t‐ROC), respectively. The net benefit was determined using decision curve analysis (DCA). Two‐sided *p* < 0.05 was deemed statistically significant.

## 3 RESULTS

### 3.1 Baseline characteristics of included patients

In this investigation, a total of 337 MIBC patients undergoing RC were enrolled (Table 2). These patients consisted of 293 (86.9%) men and 44 (13.1%) women. The mean age was 65.3 ± 9.945 years, and the mean BMI was 22.958 ± 3.001 kg/m^2^. Among all patients, 110 (32.6%) had a smoking history, 83 (24.6%) had a hypertension history, and 43 (12.8%) had a diabetes history. The median PNI was 48.8 (interquartile range [IQR]: 45.15–52.70), and the median CONUT score was 1 (IQR: 1–3). The following are body composition indicators: the median SMI was 46.92 (IQR: 41.57–52.15), the median SAI was 35.38 (IQR: 25.19–46.86), the median VAI was 36.74 (IQR: 20.06–59.27), the median SMD was 37.54 (IQR: 32.85–41.66), the median SAD was −97.57 (IQR: −103.6 to −89.21), the median VAD was −94.00 (IQR: −100.50 to −86.04), and the median VSR was 1.04 (IQR: 0.64–1.47).

**TABLE 2:** Baseline characteristics of included participants.

Characteristics	All (*n* = 337)	Characteristics	All (*n* = 337)
Gender, *n* (%)		LVI, *n* (%)	
Female	44 (13.1%)	No	262 (77.7%)
Male	293 (86.9%)	Yes	75 (22.3%)
Age, mean ± SD	65.3 ± 9.945	PNI, median (IQR)	48.8 (45.15, 52.70)
Smoking history, *n* (%)		Variant histology, *n* (%)	225 (66.8%)
No	227 (67.4%)	No	225 (66.8%)
Yes	110 (32.6%)	Yes	112 (33.2%)
BMI, mean ± SD, kg/m^2^	22.958 ± 3.0001	CONUT, median (IQR)	1 (1, 3)
Tumor diameter, *n* (%)		Positive surgical margins, *n* (%)	
<3 cm	112 (33.2%)	No	315 (93.5%)
≥3 cm	225 (66.8%)	Yes	22 (6.5%)
Tumor number, *n* (%)		Hypertension, *n* (%)	
Unifocal	207 (61.4%)	No	254 (75.4%)
Multifocal	130 (38.6%)	Yes	83 (24.6%)
pT stage, *n* (%)		Diabetes, *n* (%)	
T2	172 (51%)	No	294 (87.2)
T3	118 (35%)	Yes	43 (12.8%)
T4	47 (13.9%)	SMI, median (IQR)	46.92 (41.57, 52.15)
pN status, *n* (%)		SAI, median (IQR)	35.38 (25.19, 46.86)
pN+	62 (18.4%)	VAI, median (IQR)	36.74 (20.06, 59.27)
pN‐	275 (81.6%)	SMD, median (IQR)	37.54 (32.85, 41.66)
Peripheral nerve invasion, *n* (%)		SAD, median (IQR)	−97.57 (−103.60, −89.21)
No	281 (83.4%)	VAD, median (IQR)	−94 (−100.5, −86.04)
Yes	56 (16.6%)	VSR, median (IQR)	1.04 (0.64, 1.47)

Abbreviations: BMI, body mass index; CONUT, controlling nutritional status; IQR, interquartile range; LVI, lymphovascular invasion; PNI, prognostic nutritional index; SAD, subcutaneous adipose density; SAI, subcutaneous adipose index; SD, standard deviation; SMD, skeletal muscle density; SMI, skeletal muscle index; VAD, visceral adipose density; VAI, visceral adipose index; VSR, visceral adipose to subcutaneous adipose area ratio.

The postoperative pathological characteristics of all MIBC patients were as follows: 172 patients (51%) were in pT2 stage, 118 patients (35%) were in pT3 stage, and 47 patients (14%) were in pT4 stage. Sixty‐two patients (18.4%) were pN+, while 275 patients (81.6%) were pN‐. Out of all these patients, 225 (66.8%) exhibited tumors measuring ≥3 cm in diameter, while 130 (38.6%) had multiple tumors. Peripheral nerve invasion was detected in 56 patients (16.6%). Positive surgical margins were found in 22 patients (6.5%). Lymphovascular invasion was found in 75 patients (22.3%). In addition, 112 patients (33.2%) had variant histology subtypes (with squamous differentiation, glandular differentiation, etc.).

### 3.2 The optimal cutoff values of variables

**FIGURE 2:**
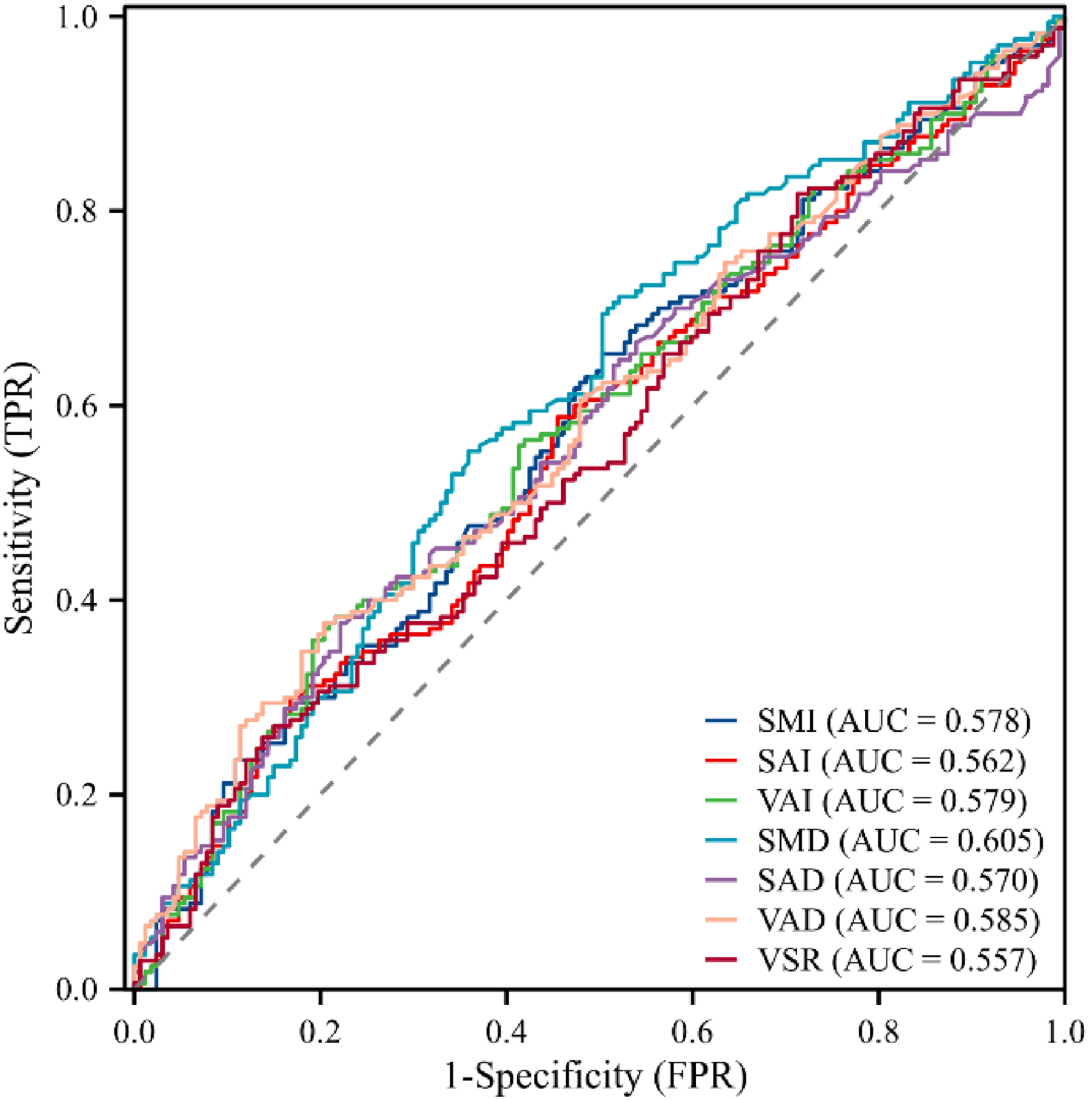
ROC of body composition and AUC. AUC, area under curve; ROC, receiver operating characteristic curve; SAD, subcutaneous adipose density; SAI, subcutaneous adipose index; SMD, skeletal muscle density; SMI, skeletal muscle index; VAD, visceral adipose density; VAI, visceral adipose index; VSR, visceral adipose to subcutaneous adipose area ratio.

The AUCs of SMI, SAI, VAI, SMD, SAD, VAD, and VSR in predicting the survival of MIBC patients after RC were 0.578 (95% CI = 0.517–0.639), 0.562 (95% CI = 0.500–0.623), 0.579 (95% CI = 0.519–0.640), 0.605 (95% CI = 0.545–0.666), 0.570 (95% CI = 0.509–0.632), 0.585 (95% CI = 0.524–0.646), and 0.557 (95% CI = 0.496–0.618), respectively (Figure 2). When Youden's index was at its highest point, the optimal cutoff values for SMI, SAI, VAI, SMD, SAD, VAD, and VSR were 48.75%, 36.57%, 21.08%, 36.46, −91.85, −87.67%, and 59.53%, respectively. Patients were divided into low SMI group (<48.75)/high SMI group (≥ 48.75), low SAI group (<36.57)/high SAI group (≥ 36.57), low VAI group (<21.08)/high VAI group (≥ 21.08), low SMD group (<36.46)/high SMD group (≥ 36.46), low SAD group (<−91.85)/high SAD group (≥ −91.85), low VAD group (<−87.67)/high VAD group (≥ −87.67), and low VSR group (<59.53%)/high VSR group (≥ 59.53%).

The low PNI group was defined as PNI < 45, whereas the high PNI group was defined as PNI ≥45.^16^ The low CONUT score group was defined as CONUT score <3, while the high CONUT score group was defined as CONUT score ≥3.^17^

### 3.3 Relevant variables for survival and survival analysis

**FIGURE 3:**
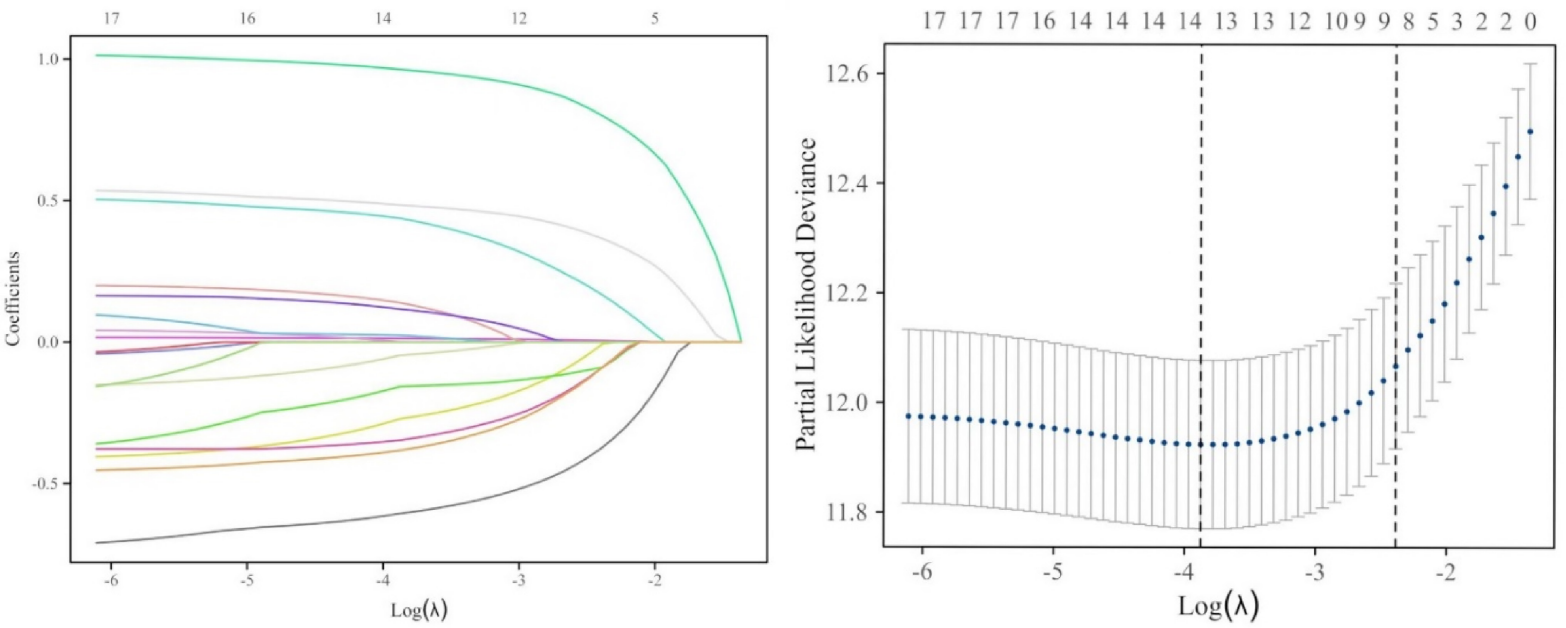
Variable selection using least absolute shrinkage and selection operator (LASSO) regression.

In univariate Cox regression analyses, 17 variables (age, BMI, pT stage, lymph node status, peripheral nerve invasion, surgical margin condition, variant histology subtypes, LVI, PNI, CONUT, SMI, SAI, VAI, SMD, SAD, VAD, and VSR) were significantly correlated with OS. The variables mentioned earlier were incorporated into the LASSO regression analysis (Figure 3). Using 10‐fold cross‐validation for screening, age, pT stage, lymph node status, surgical margin condition, variant histology subtypes, LVI, PNI, SMI, SAI, VAI, SMD, SAD, and VSR were identified as variables associated with prognosis when lambda = 0.02. These variables were considered potential predictors for OS. Multivariate Cox proportional hazards were applied to these variables for further analysis (Table 3). The results showed pT stage (HR = 1.669, 95% CI = 1.189–2.343, *p* = 0.003), positive lymph node (HR = 2.720, 95% CI = 1.851–3.996, *p* < 0.001), LVI (HR = 1.694, 95% CI = 1.157–2.482, *p* = 0.007), PNI (HR = 0.514, 95% CI = 0.368–0.717, *p* < 0.001), and SMD (HR = 0.660, 95% CI = 0.460–0.947, *p* = 0.024). Therefore, pT stage, positive lymph nodes, LVI, PNI, and SMD are independent risk factors for the survival of patients following RC.

**TABLE 3:** Results of univariate and multivariate analyses of factors associated with overall survival after LASSO regression screening.

Characteristics	T (*N*)	Univariate analysis		Multivariate analysis	
		HR (95% CI)	*p*	HR (95% CI)	*p*
Age	337	1.030 (1.014–1.046)	< 0.001	1.015 (0.998–1.033)	0.081
pT stage	337		< 0.001		**0.003**
T2	172	Reference		Reference	
>T2	165	2.550 (1.864–3.487)	< 0.001	1.669 (1.189–2.343)	
pN status	337		< 0.001		**< 0.001**
pN‐	275	Reference		Reference	
pN+	62	3.789 (2.705–5.308)	<0.001	2.720 (1.851–3.996)	
Surgical margins	337		0.014		0.422
Negative	315	Reference		Reference	
Positive	22	2.041 (1.216–3.426)	0.007	1.247 (0.728–2.136)	
Variant histology	337		0.001		0.325
With	112	Reference		Reference	
Without	225	0.599 (0.441–0.813)	0.001	0.850 (0.614–1.175)	
LVI	337		< 0.001		**0.007**
Without	262	Reference		Reference	
With	75	2.553 (1.843–3.536)	< 0.001	1.694 (1.157–2.482)	
PNI	337		< 0.001		**< 0.001**
<45	80	Reference		Reference	
≥45	257	0.482 (0.350–0.664)	< 0.001	0.514 (0.368–0.717)	
SMI	337		0.006		0.642
<48.75	195	Reference		Reference	
≥48.75	142	0.646 (0.471–0.887)	0.007	0.922 (0.654–1.299)	
SAI	337		0.008		0.091
<36.57	161	Reference		Reference	
≥36.57	176	1.505 (1.109–2.043)	0.009	1.405 (0.947–2.085)	
VAI	337		0.001		0.504
<21.08	244	Reference		Reference	
≥21.08	93	1.703 (1.245–2.331)	< 0.001	1.190 (0.713–1.986)	
SMD	337		< 0.001		**0.024**
<36.46	154	Reference		Reference	
≥36.46	183	0.598 (0.442–0.809)	< 0.001	0.660 (0.460–0.947)	
SAD	337		0.004		0.828
<−91.85	236	Reference		Reference	
≥−91.85	101	1.593 (1.167–2.174)	0.003	1.048 (0.687–1.598)	
VSR	337		0.004		0.051
≥59.53	270	Reference		Reference	
<59.53	67	1.686 (1.195–2.379)	0.003	1.574 (0.997–2.485)	

Abbreviations: CI, confidence interval; HR, hazard ratio; LASSO, least absolute shrinkage and selection operator; LVI, lymphovascular invasion; PNI, prognostic nutritional index; SMI, skeletal muscle index; SAI, subcutaneous adipose index; VAI, visceral adipose index; SMD, skeletal muscle density; SAD, subcutaneous adipose density; VAD, visceral adipose density; VSR, visceral adipose to subcutaneous adipose area ratio.

These *p*‐values less than 0.05 are statistically significant.

**FIGURE 4:**
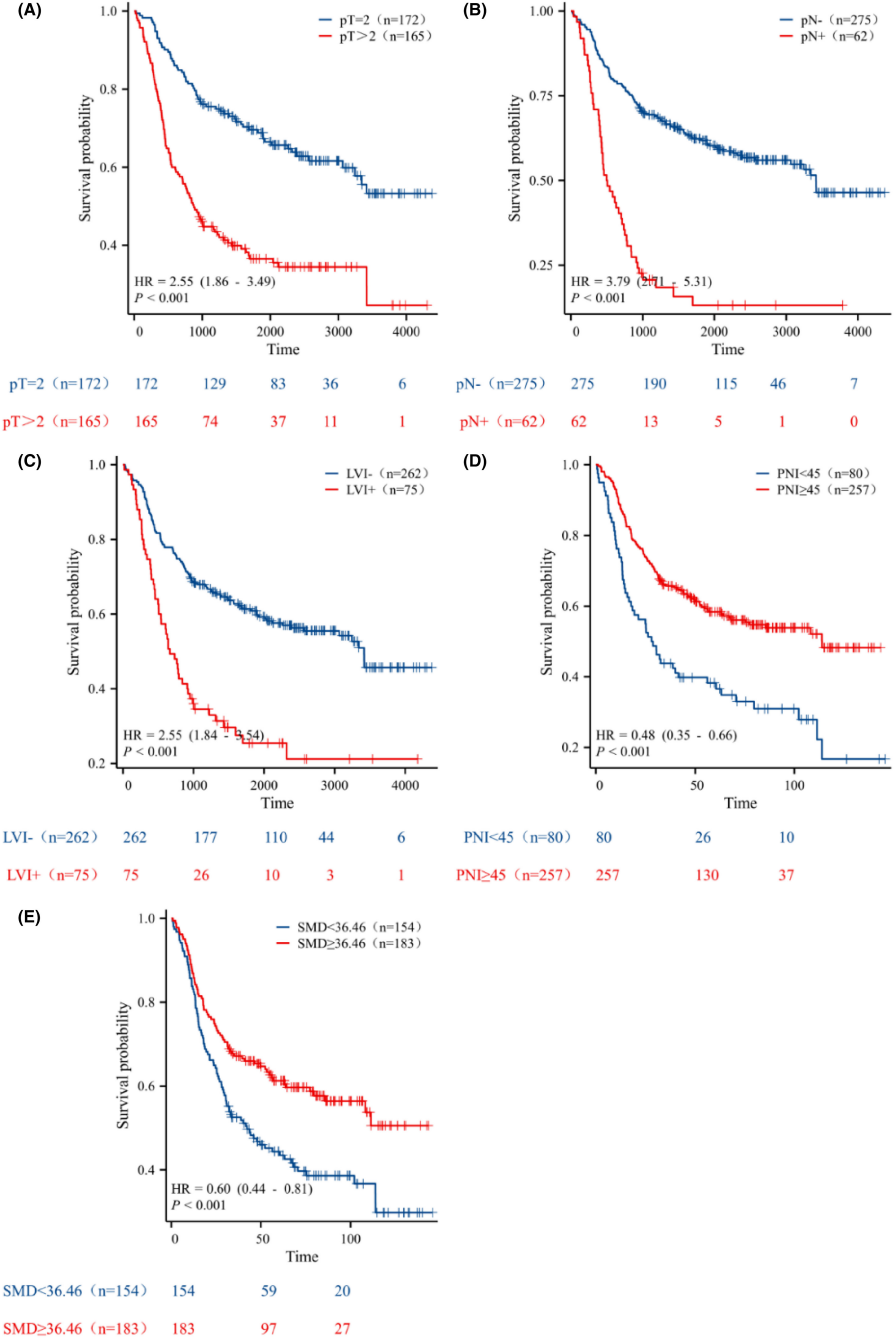
Kaplan–Meier survival curves for pT stage (A), lymph node status (B), lymphovascular invasion (C), PNI (D), and SMD (E).

The median duration of follow‐up was 46 (IQR: 18–81) months. During the follow‐up, a total of 170 patients (170/337, 50.45%) died, and the 1‐, 3‐, and 5‐year cumulative OS rates were 85.16%, 59.20%, and 46.50%, respectively. Kaplan–Meier curves showed that the OS of the pT2, pN‐, without LVI, high PNI, and high SMD groups was significantly higher than that of the pT >2, pN+, with LVI, low PNI, and SMD groups (all *p* < 0.001), as depicted in Figure 4. A higher T stage, positive lymph nodes, LVI, a low PNI, and a low SMD were associated with decreased OS.

### 3.4 Construction and validation of the nomogram

**FIGURE 5:**
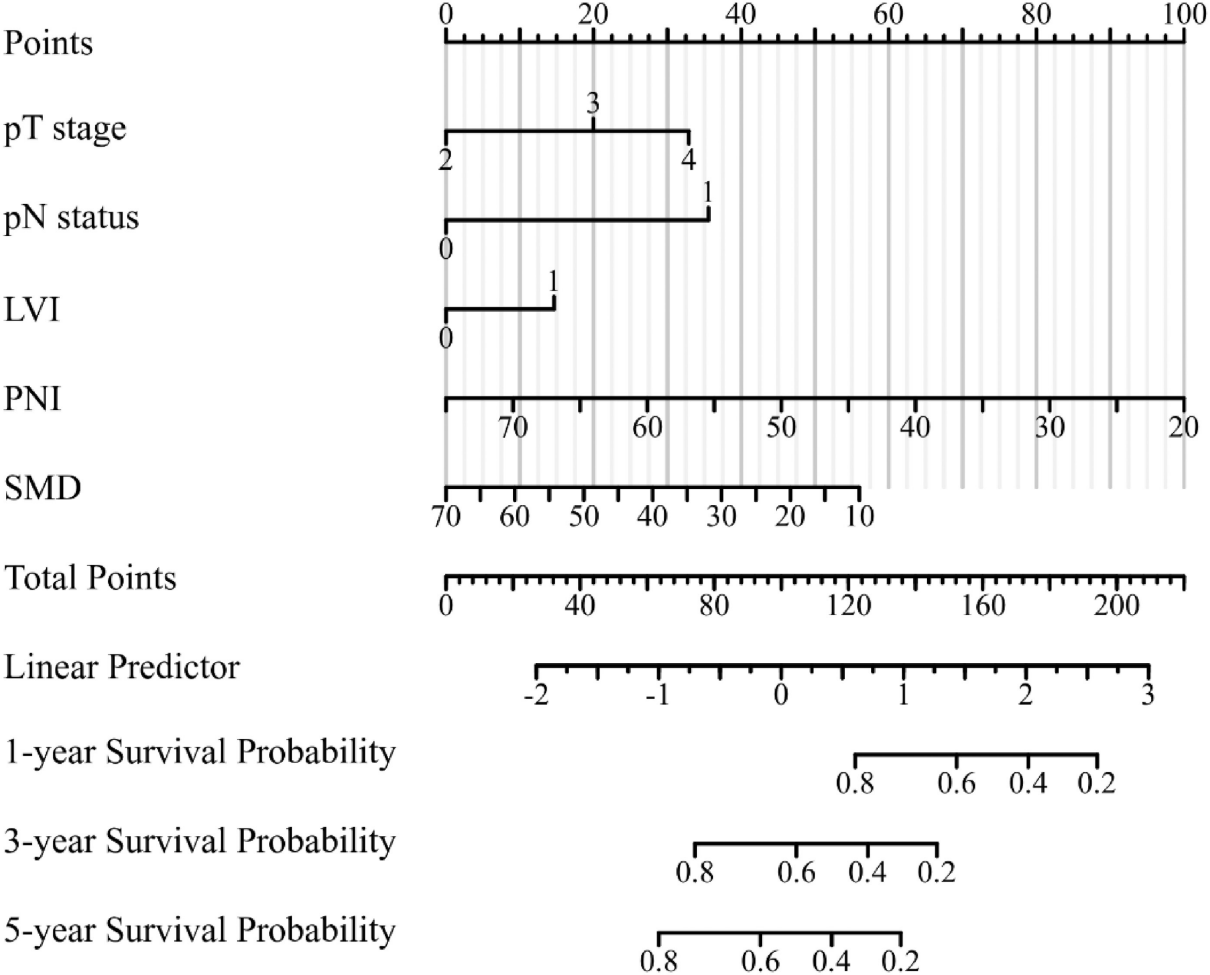
Nomogram for predicting 1‐, 3‐, and 5‐year survival of MIBC patients undergoing radical cystectomy.

A nomogram was created by utilizing the risk factors (pT stage, lymph node status, LVI, PNI, and SMD) determined through multivariate Cox regression analyses. The nomogram was built using five‐point scales for variables, and the total points were determined by summing them. Figure 5 displays the survival probabilities for 1, 3, and 5 years by linking the total point axis to the three outcome axes using a vertical line.

**FIGURE 6:**
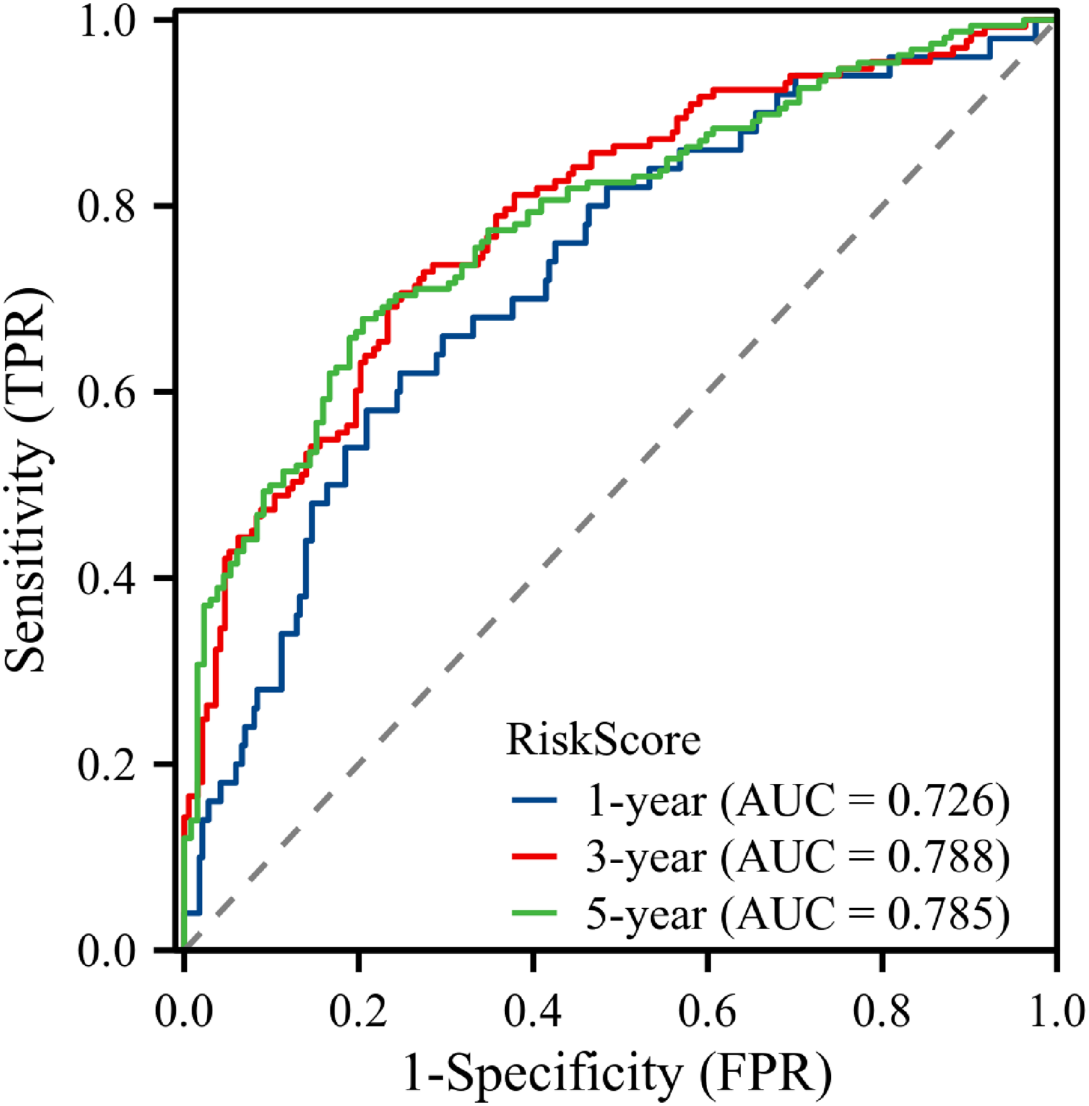
Time‐dependent receiver operating characteristic of the nomogram.

**FIGURE 7:**
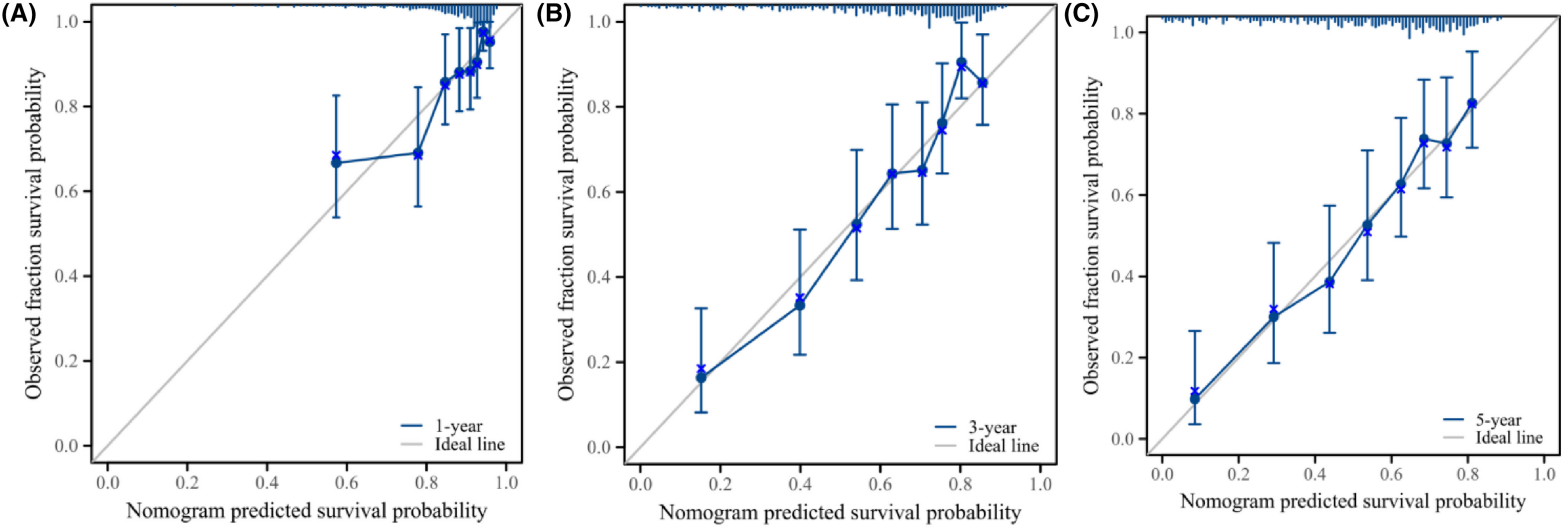
Calibration plot of the nomogram for predicting 1‐year OS (A), predicting 3‐year OS (B), and 5‐year OS (C).

**FIGURE 8:**
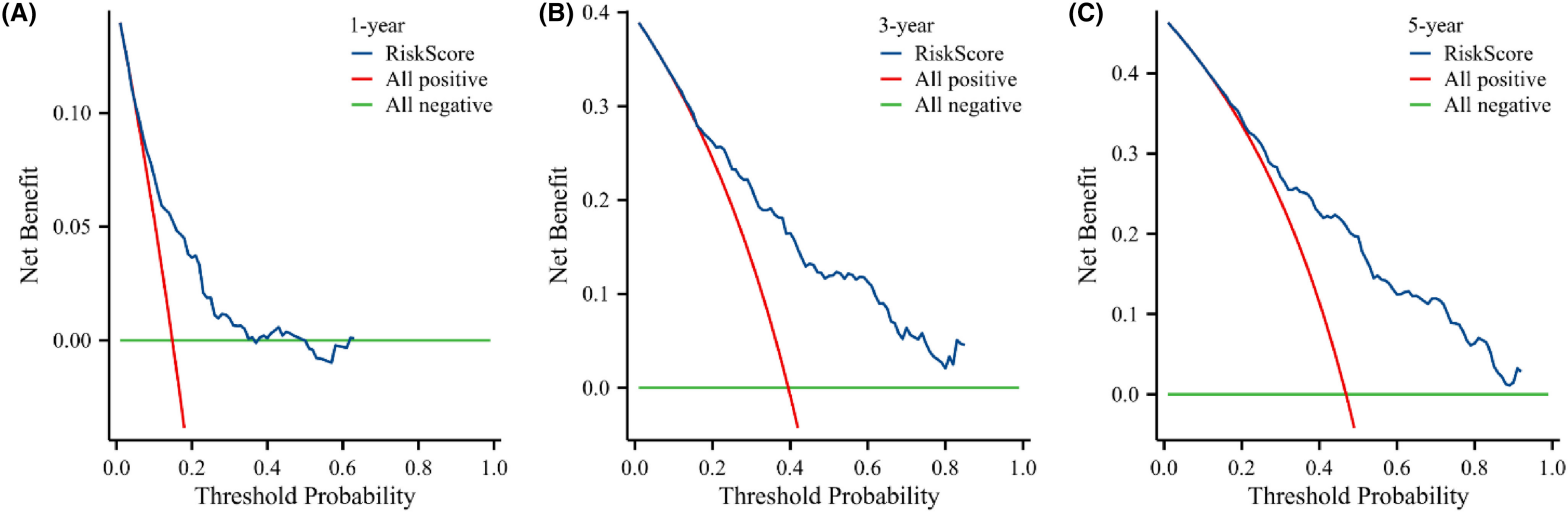
Decision curve analysis of the nomogram for predicting 1‐ year OS (A), 3‐ year OS (B), and 5‐ year OS (C).

In this study, the prediction model was validated using the bootstrap resampling technique. The model's C‐index was 0.714 (95% CI: 0.695–0.733), indicating adequate discrimination. The t‐ROC curve was further drawn, and the AUCs of the model for 1‐, 3‐, and 5‐year OS were 0.726, 0.788, and 0.785 (Figure 6), respectively, revealing that the nomogram has good prediction performance. The calibration plot for the 1000‐resample bootstrap validation model was comparable to the actual value, especially for the 3‐year OS (Figure 7). Decision curve analysis (DCA) was used to evaluate the clinical benefit to the patients. Figure 8 demonstrates that the decision curves were always greater than the “All negative” and “All positive” lines, indicating the clinical applicability of the model.

## 4 DISCUSSION

Even after RC has been performed, the prognosis of MIBC patients is highly variable.^18^ The TNM staging system is currently the most popular instrument for predicting the individual survival of patients, but its precision is limited.^6^ Over the past few years, there have been several described prognostic models; however, their excessive complexity and limited applicability render them inappropriate for clinical utilization.^19^, ^20^, ^21^ Therefore, a concise and accurate model to predict the prognosis of MIBC patients undergoing RC is urgently needed.

In this study, we evaluated the prognostic value of body composition and nutritional indicators in a large cohort of MIBC patients undergoing RC and found that patients with a lower PNI and SMD had inferior OS. Low PNI, high CONUT, low SMI, high SAI, high VAI, low SMD, high SAD, high VAD, and high VAR were significantly correlated with poor OS in the univariate Cox proportional hazard model. However, after LASSO regression and multivariate analyses, it was found only that low PNI and low SMD were independent prognostic factors for OS. Additionally, pT stage, lymph node status, and LVI were also independent predictors of OS. Based on the aforementioned findings, we further constructed a novel nomogram to predict the prognosis of MIBC patients. It was anticipated that the model's accuracy and excellent prediction performance would play a significant role in clinical decision‐making.

Previous research has demonstrated that body composition and nutritional status have substantial influences on the prognosis of malignant tumors.^7^, ^11^, ^14^ Body composition parameters (SMI, SAI, VAI, SMD, SAD, VAD, and VSR) can be obtained via CT imaging at the third lumbar vertebral level.^22^ Patients with the same TNM stage may have various outcomes if their visceral fat, subcutaneous fat, and skeletal muscle contents and proportions differ. Zheng et al. demonstrated that alterations in skeletal muscle mass have an important impact on the prognosis of patients with gastric cancer following radical surgery.^9^ Zhuang et al. discovered that skeletal muscle mass was an independent prognostic factor for OS in head and neck cancer patients.^23^ Similarly, Sanchez et al. showed that body composition can be used as a novel prognostic factor with the potential to enhance bladder cancer patients' survival.^24^ The findings of this research align with prior studies, indicating that SMD can serve as a standalone prognostic indicator for patients with MIBC. The loss of skeletal muscle tissue in tumor patients is primarily attributable to the active systemic inflammatory response in tumor patients, the increase in reactive oxygen species, the mitochondrial dysfunction caused by oxidative stress, and the dysfunction of skeletal muscle cells in the tumor microenvironment.^25^, ^26^, ^27^ In addition, skeletal muscle tissue decreases, as does its secretion of antitumor myokines, resulting in a worse prognosis for cancer patients.^28^ Malnourished patients with malignant tumors frequently exhibit the aforementioned physiologic alterations, so SMD can indicate prognosis.

PNI is frequently used as a nutritional status indicator, which can effectively predict the prognosis of MIBC patients after RC.^19^ It has been proven to be a prognostic factor for a variety of tumors, which is evaluated by serum albumin and lymphocytes.^11^, ^16^, ^29^ Albumin plays a role in transporting and maintaining plasma colloidal osmotic pressure and is frequently used to assess nutritional status.^30^ As the primary immune cells, lymphocytes can stimulate the immune response in the tumor microenvironment and release tumor necrosis factor, interferon‐γ and other cytokines, producing antitumor effects.^11^ Consequently, a low PNI indicates malnutrition and systemic immune dysfunction, both of which are associated with a poor prognosis in MIBC patients.

At present, the effect of concurrent body composition and PNI measurements in predicting prognosis in patients with MIBC is still understudied. In this large cohort study, we found that PNI and SMD were independent predictors of MIBC. Furthermore, we consolidated the five selected characteristics (pT stage, lymph node status, LVI, SMD, and PNI) into the model through multivariate analyses and then obtained a nomogram with excellent predictive performance. To date, this is the first nomogram that combines body composition and nutritional indicators to predict the prognosis of MIBC patients. Compared with other nomograms, this nomogram has the advantages of simplicity, accuracy, and excellent prediction performance. Moreover, this study is the result of a large population and long‐term follow‐up. In addition, all of the clinical parameters needed by this nomogram are accessible through preoperative examination and routine pathologic examination. Using this point‐based nomogram, both clinicians and patients could calculate the individual probability of survival with ease.

Despite the fact that we successfully developed and validated a nomogram to predict the individual survival probability of MIBC patients undergoing RC, our study did have several limitations. First, due to the retrospective design with a single center, selection bias is inevitable. Second, this nomogram lacks external validation, and the efficacy of this nomogram in an external cohort is ambiguous. Third, the impact of postoperative changes in body composition on prognosis was not evaluated. In addition, the potential influence of neoadjuvant and adjuvant chemotherapy on prognosis, which may affect overall survival, remains unexplored. In response to the aforementioned issues, we will further collect prospective cohort information from our center for verification. We will also conduct multicenter, large‐sample research to try to fit prediction models and better guide clinical practice.

## 5 CONCLUSION

To summarize, we developed an innovative nomogram that utilizes body composition and nutritional markers to forecast the outcome of MIBC patients undergoing RC. Several techniques were employed to verify the performance of this nomogram, and the findings demonstrated the outstanding precision, dependability, and usefulness of the nomogram in forecasting.

## AUTHOR CONTRIBUTIONS

**Xin Zhong:** Data curation (equal); formal analysis (equal); investigation (equal); writing – original draft (equal). **Yunzhe Pan:** Data curation (equal); formal analysis (equal); software (equal); writing – original draft (equal). **Kang Wu:** Data curation (equal); formal analysis (equal); visualization (equal); writing – original draft (equal). **Langkun Wang:** Investigation (equal); software (equal). **Peng Dou:** Investigation (equal); software (equal). **Ping Tan:** Methodology (equal); validation (equal). **Peng Zhang:** Conceptualization (equal); supervision (equal); writing – review and editing (equal). **Xiang Li:** Conceptualization (equal); writing – review and editing (equal).

## FUNDING INFORMATION

The study was supported by the Science and Technology Program of Sichuan Science and Technology Department (2023YFS0315).

## CONFLICT OF INTEREST STATEMENT

There are no competing interests disclosed by the authors.

## ETHICS STATEMENT

The Ethics Committee of West China Hospital, Sichuan University, reviewed and authorized the study. The study participants gave their written consent to take part in this research.

## Data Availability

The corresponding author will provide the original data supporting the conclusions of this article upon a justifiable request.
